# The Emerging Role of Computed Tomography Coronary Angiography in the Left Main Stem Percutaneous Coronary Intervention

**DOI:** 10.31083/RCM37379

**Published:** 2025-05-26

**Authors:** Asad Shabbir, Abdelrahman Attia, Nikkole Marie Weber, Mustafa Alhassan, Monika Radike, Conor M Lane, Apurva Bhavana Challa, Malgorzata Wamil

**Affiliations:** ^1^John Radcliffe Hospital, Oxford University Hospitals, OX3 9DU Oxford, UK; ^2^Cardiology Department, Great Western Hospital NHS Trust, SN3 6BB Swindon, UK; ^3^Radiology Department, Mayo Clinic Rochester, Rochester, MN 55903, USA; ^4^Radiology Department, Liverpool Heart and Chest Hospital NHS Foundation Trust, L14 3PE Liverpool, UK; ^5^Cardiology Department, Mayo Clinic Healthcare, W1B 1PT London, UK

**Keywords:** computed tomography coronary angiography, computed tomography derived fractional flow reserve, left main stem percutaneous coronary intervention, coronary artery disease, plaque characterisation

## Abstract

Recent advancements in computed tomography have significantly transformed the clinical application of this technique in diagnosing and managing coronary artery disease (CAD). Computed tomography coronary angiography (CTCA) has emerged as a leading non-invasive imaging modality, often serving as the first-line investigation to exclude obstructive CAD in patients with chronic coronary syndrome. Beyond its utility in diagnosing CAD, CTCA has become instrumental in procedural planning for percutaneous coronary intervention (PCI), particularly in complex cases such as left main stem (LMS) interventions, where peri-procedural risks are elevated. This review highlights the evolving role of CTCA in LMS PCI, underscoring its clinical utility in improving procedural precision and, subsequently, patient outcomes. Recent technological advancements, including detailed multiplanar and three-dimensional (3D) reconstructions, CT-derived fractional flow reserve (CTFFR), and the integration of artificial intelligence (AI) algorithms, have expanded the capabilities of CTCA. These innovations allow for comprehensive anatomical and functional assessments, enabling precise plaque morphology, lesion complexity, and bifurcation anatomy evaluations alongside PCI simulations. By offering detailed insights into coronary vasculature and lesion characteristics, CTCA provides critical information for optimising LMS PCI strategies. This review explores the current applications and future potential of CTCA in guiding LMS PCI, highlighting its role in improving procedural planning, risk assessment, and overall management of this challenging patient population.

## 1. Introduction

Computed tomography (CT) is widely used to assess stable chest pain in patients with chronic coronary syndrome (CCS). It is a sufficiently reliable method for assessing obstructive coronary artery disease (CAD) and is now an established alternative to invasive coronary angiography (ICA). Clinical studies have rigorously evaluated the safety of computed tomography coronary angiography (CTCA) in this context, supporting its use in clinical practice (Table 1, Ref. [1, 2, 3, 4, 5, 6, 7, 8, 9, 10, 11, 12, 13, 14, 15, 16, 17, 18]) [1, 19].

**Table 1.** **The role of computed tomography coronary angiography (CTCA) in planning percutaneous coronary interventions (PCI) with a focus on left main stem (LMS) disease**.

Trial Name	Author	Study Type	Population	Number of patients	LMS disease	Objective	Key Outcomes	Conclusion/Relevance
CATCH (2013) [2]	Linde, Jesper James *et al*.	Randomized Control	Suspected ACS with normal ECG and negative troponin	600 (299 CTCA, 301 functional testing)	N/S	Evaluate the impact of CTCA on the referral rate for ICA	No significant increase in ICA rate. CTCA improved PPV for detection of CAD, increased the frequency of revascularization	CTCA improves PPV for the detection of significant CAD when compared with functional imaging
PROMISE (2015) [3]	Douglas, Pamela S. *et al*.	Randomized Control	Stable CAD	10,003 (4996 CTCA, 5007 functional testing)	N/S	Compare CTCA vs functional testing	CTCA vs functional testing showed no significant difference in clinical outcomes	CTCA is a viable alternative to functional testing
CAD-MAN (2016) [4]	Dewey *et al*.	Randomized control	Suspected CAD referred for ICA	329 (167 CTCA, 162 ICA)	N/S	Evaluate whether ICA or CTCA should be performed in patients with intermediate pre-test probability as compared to ICA	CTCA reduced the need for ICA, and added a greater diagnostic yield from ICA. Low major procedural complications in both groups. Less minor complications in CTCA group with shorter hospital stay	CTCA can be effectively used as a gatekeeper for ICA
SCOT-HEART (2018) [5]	SCOT-HEART Trial Investigators	Randomized Control	Suspected CAD	4146 (2073 CTCA, 2073 standard care)	N/S	Assess the effect of CTCA on 5-year clinical outcome	Adding CTCA to standard care resulted in a lower composite endpoint of CVD or non-fatal MI. No significant increase in ICA rates	CTCA can add a prognostic benefit to patients with stable angina.
SYNTAX III Revolution (2018) [6]	Collet, Carlos *et al*.	Randomized Control	Three-vessel CAD or LMS disease	223 (112 CTCA, 111 ICA )	25 (11%)	Assess the usefulness of CTCA in 3VD or LMs disease	High agreement in decision-making based on CTCA and ICA. CTFFR can influence and alter decision	CTCA can be used to decide the revascularization strategy PCI vs CABG without the need for ICA
CONSERVE (2019) [7]	Chang, Hyuk-Jae *et al*.	Randomized Control	Stable CAD referred for ICA	1611 (808 ICA, 823 CTCA)	N/S	Assess safety and diagnostic yield of a selective referral strategy using CTCA compared with a direct referral strategy using ICA as the index procedure	No significant difference in MACE. Lower rate of ICA in CTCA group with less revascularization, and greater diagnostic yield	CTCA can safely and effectively be used as a first line for diagnosis of CAD, also it increases the diagnostic yield of ICA
FORECAST (2021) [8]	Curzen, Nick *et al*.	Randomized control	Stable CAD	1400 (700 CTCA + CTFFR, 700 standard care)	N/S	Test whether an evaluation strategy based on CTFFR would improve economic and clinical outcomes compared with standard care	CTCA with selective CTFFR in stable angina did not differ significantly from standard clinical care pathways in cost or clinical outcomes but did reduce the use of invasive coronary angiography	CCTA with CTFFR may not reduce cost, but it can reduce the need for ICA.
ISCHEMIA (sub-analysis) (2022) [9]	Bangalore, Sripal *et al*.	Post-hoc sub-analysis of ISCHEMIA trial)	LMS disease patients in ISCHEMIA trial	3699 (962 with intermediate LMS disease, 2737 with no intermediate LMS disease)	3699 (100%)	Compare the clinical and quality-of-life (QoL) outcomes between invasive and conservative approaches in patients with intermediate LMS disease on CTCA	There was no significant difference between an invasive and conservative strategy for the primary and secondary outcomes. The invasive strategy increased procedural MI, reduced nonprocedural MI, and improved angina-related quality of life	CTCA can distinguish and quantify significant LMS disease.
DISCHARGE (2022) [1]	DISCHARGE Trial Group	Randomized Control	Stable CAD with an intermediate pre-test probability of obstructive disease	3561 (1808 CTCA vs 1753 ICA)	N/S	Compare the effectiveness of CTCA and ICA in the management of CAD	Similar MACE events in both groups. Higher procedure-related complications in the ICA group	CTCA may offer a safer diagnostic approach when compared to ICA with similar rates of MACE
Kawashima *et al*. (2022) [10]	Kawashima, Hideyuki *et al*.	(Post-hoc sub-analysis of SYNTAX III REVOLUTION trial)	3VD or LMS	183	47 (25.7%)	investigate the correlation and agreement between QFR and CTFFR in patients with 3VD or LMS disease	There was a strong correlation between QFR and CTFFR. Highest diagnostic concordance was found in RCA disease	CTFFR is a useful non-invasive tool for the assessment of complex CAD
RAPID-CTCA (2022) [11]	Gray, Alasdair J *et al*.	Randomized Control	Patients with suspected ACS	1748 (877 CTCA-guided strategy, 871 standard care)	N/S	Investigate the role of CTCA in patients with suspected ACS	CTCA did not alter overall interventions or 1-year clinical outcomes, but it did increase hospital stay and costs	CTCA should not be used routinely in ACS patients
Bypass-CTCA (2023) [12]	Jones, Daniel A., *et al*.	Randomized Control	Patients with previous CABG referred for ICA	688 (321 in CTCA + ICA group, 342 ICA alone)	N/S	See if the use of CTCA in patients with previous CABG can make ICA quicker and safer	CTCA lead to a reduction in procedure time, CIN, improved patient satisfaction, and a lower complication rate	CTCA should be considered before ICA in patients with a history of CABG
ADVANCE (2020) [13]	Patel, Manesh R *et al*.	Prospective, multicentre	Patients who underwent CTCA for assessment of CAD	5083	N/S	Evaluate the relationship between CTFFR and downstream care and clinical outcomes	Determine sex-based differences in the rate of ICA, incidence of nonobstructive CAD, and revascularization rates at 90 days	Lower death, lower MI, and a trend toward lower MACE with negative CTFFR. CTFFR changed recommendation in 63% of subjects as compared to CCTA alone, fewer negative ICA, and predicted revascularization.
CREDENCE (2020) [14]	Stuijfzand, Wijnand J *et al*.	Prospective comparative	Patients undergoing non-emergent invasive angiography	612 patients	N/S	Compare the diagnostic accuracy of CTCA combined with CTFFR vs functional imaging in estimating vessel-specific FFR using invasive FFR as a reference	Individual comparisons of APCs or CTFFR to MPI vessel-specific perfusion deficits. Post-PCI FFR prediction by CTFFR ‘virtual stenting’	CTFFR is superior to perfusion imaging in the assessment of obstructive and non-obstructive atherosclerotic plaques
The NXT trial (2014) [15]	Nørgaard, Bjarne L *et al*.	Prospective	Stable CAD undergoing CTCA + CTFFR before ICA	254	N/S	Determine the diagnostic performance of CTFFR vs Invasive FFR	Diagnostic accuracy, sensitivity, specificity, PPV, NPV of CTFFR	CTFFR was highly diagnostic of hemodynamically significant CAD when compared to invasive FFR
Gaur *et al*. (2016) [16]	Gaur, Sara, *et al*.	(sub-study from NXT trial)	Stable CAD undergoing CTCA before ICA	254	N/S	Evaluate the association between coronary stenosis severity, plaque characteristics, CTFFR, and lesion-specific ischemia identified by FFR	none	Stenosis severity, plaque characteristics, and CTFFR predict lesion-specific ischemia. Plaque assessment and CTFFR provide improved discrimination of ischaemia compared with stenosis assessment alone.
Van Mieghem *et al*. (2006) [17]	Van Mieghem, Carlos A G *et al*.	Prospective	Patients who had previous PCI to LMs awaiting follow-up ICA	74	74 (100%)	Diagnostic performance of CTCA to detect ISR after stenting of LMS	none	The accuracy of CTCA for the detection of angiographic ISR was 93%. High sensitivity, specificity, and positive and negative predictive values. Lower accuracy for bifurcation lesions
Ko *et al*. (2024) [18]	Ko, Brian *et al*.	Prospective Sub-analysis of P3 trial	Stable CAD and invasive FFR <0.8 who had both CTCA and OCT pre-pci	65 patients (65 vessels)	P3 trial excluded left main disease	Establish the usefulness of CTCA in guiding PCI and stent sizing	none	No proportional or systemic differences. Substantial agreement in stent sizing between OCT and CTCA

LMS, left main stem; ACS, acute Coronary Syndrome; ECG, electrocardiogram; CTCA, computed tomography coronary angiography; ICA invasive coronary angiography; PPV, positive predictive value; CAD, coronary artery disease; 3VD, three-vessel disease; CTFFR, computed tomography derived fractional flow reserve; FFR, fractional flow reserve; N/S, not stated; CABG, coronary artery bypass grafting; MACE, major adverse cardiovascular events; QFR, quantitative flow ratio. Table 1 provides a structured literature overview, emphasising CTCA’s evolving role in PCI planning. It illustrates CTCA’s evidence-based advantages for identifying anatomical and functional challenges, predicting outcomes, and enhancing procedural precision. The table includes the column ‘LMS disease’, which specifies whether LMS cases were included in the study population. Although few studies specifically included LMS cases, we present a systematic review of how CTCA supports PCI strategy decision-making across clinical contexts. Table 1 highlights randomised controlled trials that evaluate CTCA’s utility in various populations, with attention to studies that explicitly included LMS patients, as stated in the column ‘LMS disease’. By reviewing the presence or absence of LMS-specific data in the trials, this section examines the generalisability of the findings to LMS populations. It provides insight into CTCA’s potential to enhance procedural planning for LMS PCI. The table also presents the evidence behind the role of CTFFR compared to invasive assessment and the use of functional imaging. It highlights the number of patients with LMS included in those studies. Lastly, it summarises key evidence emphasising the role of CTCA in post-PCI LMS stent stenosis.

Traditionally, the benefit of CTCA over ICA has been that the non-invasive assessment is associated with a significantly lower risk [20], given that the associated hazard of invasive coronary vessel manipulation can be largely excluded. However, there has been significant development of CTCA technology in the last decades, such that far greater information can be obtained beyond solely an axial anatomical assessment of the coronary vessels [21].

Progressive iterative developments of CT scanners and the software used for data analysis have led to significant technological advancement in recent years. Multiplanar and three-dimensional (3D) reconstructions of the vessels are now readily available as part of almost all CTCA analysis software packages. This enables an accurate reconstruction of the vessel architecture and a detailed analysis of the vessel lumen contours and stenosis with the vessel in profile. This offers insight into vessel tortuosity and bifurcation characteristics, which provide essential information to assist with percutaneous coronary intervention (PCI) [22]. Plaque morphology, distribution, and degree of calcification are also helpful to understand in advance of ICA, as these allow the operator to gain a deeper understanding of which plaque modification techniques are likely to be required, including advanced calcium treatment techniques such as rotablation and intravascular lithotripsy (IVL) [23]. CT-derived physiology, such as those that derive fractional flow reserve from CT (CTFFR), are reliable and valuable functional assessments that can also facilitate coronary PCI planning with simulation of stent placement, allowing for the prediction of stent results [24]. CTCA also enables the precise targeting of PCI, contributing to a reduction in both radiation exposure and contrast usage. Finally, the subtended mass of the myocardium can be derived from CT data, which assists with bifurcation decisions and whether a side-branch coronary wire might be required during PCI for vessel protection and/or re-entry [25].

ICA remains the preferred imaging modality for evaluating left main stem (LMS) stenosis, but interpretation can be challenging. Multi-slice CT has a sensitivity of 95% and a specificity of 98% for detecting significant lesions in the LMS [26]. Patients with LMS disease are a particularly challenging subset, as the peri-procedural complication rate is higher than that of non-LMS PCI. Most LMS interventions are complex in that they often involve advanced plaque modification and two major bifurcation vessels: the left anterior descending vessel (LAD) and the left circumflex vessel (LCx). Similarly to structural intervention for valvular heart disease, gaining as much anatomical information as possible before undertaking complex interventions can significantly assist the operator during the case. Although the use of CT in LMS PCI planning has yet to be shown to be beneficial from a peri-procedure mortality perspective, the additional CT data can support the operator in understanding the nature of the LMS lesion, which PCI techniques might be required and resolve, at least in part, anatomical peri-procedural ambiguity. This review highlights recent advancements in CTCA, focusing on its application in LMS PCI.

## 2. Aortic and Coronary Anatomical Variation by CT

CT has exceptional sensitivity for identifying aortic and coronary anatomy variations, specifically relating to the LMS. Observational studies have demonstrated many anatomical variations in the configuration of the LMS ostium and its position in the aortic root [27]. These studies have afforded a deeper appreciation of the LMS’s anatomical variations, especially concerning sex differences in vessel dimensions, with a mean LMS ostium of approximately 5.0–5.5 mm in males and 4.5–5.0 mm in females [27]. Other common variants of LMS anatomy are short or very short LMS shafts where the LAD and LCx open into the aortic root. A misleading appearance of a very-long LMS shaft can be associated with an aberrant ostial LCx origin, and the assumed LMS shaft is, in fact, the LAD.

**Fig. 1.**
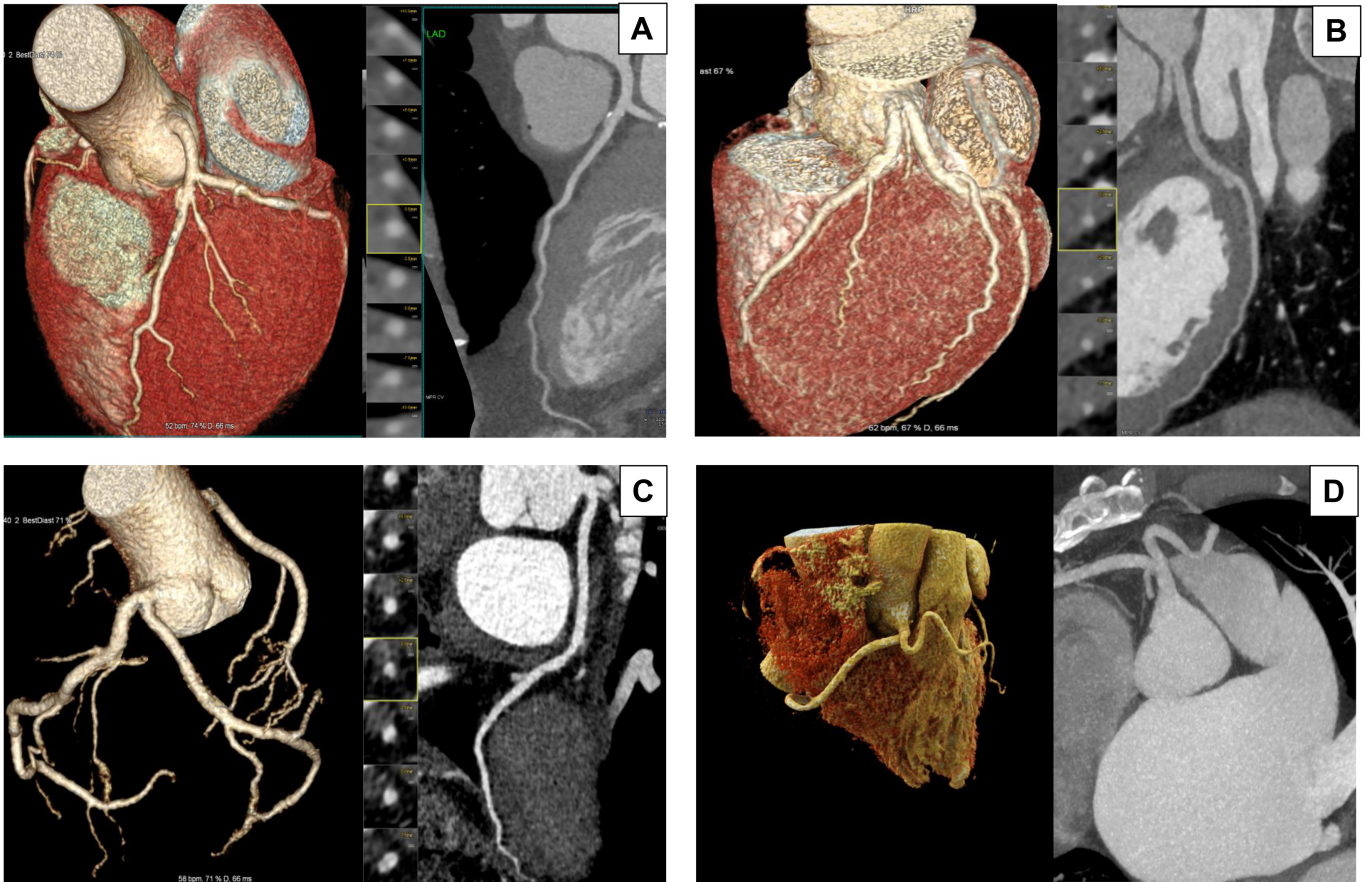
**Anatomical variations of the Left Main Stem (LMS)**. This figure illustrates four anatomical variations of the LMS by CTCA. In each panel, the three-dimensional (3D) volume-rendered image on the right and the image on the left show curved multiplanar reconstruction. (A) Typical bifurcation: The LMS originates from the left coronary sinus and bifurcates into the left anterior descending (LAD) and left circumflex (LCx) arteries. This configuration is the most common anatomical presentation. (B) Absent LMS: The LAD and LCx arteries arise separately from the left coronary sinus, resulting in the absence of a unified LMS. This variation is observed in approximately 0.41% of the population. (C) Short LMS: The LMS has a shorter course before bifurcating into the LAD and LCx arteries. A short LMS is generally defined as measuring less than 5 mm long. (D) Anomalous origin from the right coronary sinus: The LMS anomalously originates from the right coronary sinus and courses between the aorta and pulmonary artery. This rare anomaly is clinically significant due to its association with myocardial ischemia and sudden cardiac events. Understanding these anatomical variations is crucial for planning of LMS intervention. Images were acquired using a Siemens Somatom Force computed tomography (CT) scanner.

Angulation can also vary significantly, which can greatly impact the success of PCI. Although the shaft of the LMS vessel is almost straight, the bifurcation angle of the LAD and LCx is of critical importance in understanding LMS PCI. This is relevant when attempting to deliver devices to the vessels and selecting bifurcation strategies whereby bifurcation angles directly dictate which technique would be performed [28, 29]. The profile of the vessel, assessed by CT, is also helpful. Generally, coronary vessels become narrower as they bifurcate as side branches. As a result, the vessel profile becomes smaller and more distal from the aorta. Whilst the same can be said for the LMS, there are anatomical variations of the LMS shaft that CTCA can readily identify. These are classified into bi-concave shapes, tapering, combined type (i.e., bi-concave and tapering), cone-shaped, and funnel-shaped [27]. The anatomical variation of the LMS and the orientations of the LMS in the aortic root are shown in Fig. 1.

## 3. The Use of CT in Left Main Stem PCI Planning

With multiplanar and 3D reconstructions, most software packages allow the on-the-fly vessel assessment with virtual modelling. This enables the vessel to be interrogated on a workstation and allows the operator to find optimal angiographic projections offline. This may save contrast by manipulating an image on a workstation, thereby negating multiple contrast injections during the PCI procedure. This is especially useful in LMS PCI, where procedures are generally longer and require more contrast. Furthermore, in some patients with ambiguous invasive angiographic bifurcation anatomy of the LAD and LCx, and sometimes an Intermediate branch, the offline assessment of projections can lay out the bifurcation angle precisely and allow for significant forethought of the LMS bifurcation PCI strategy and technique in advance of the PCI procedure [30]. At times, angiographic projections can be misleading with regard to bifurcation angles, and CT bypasses this flaw. Essentially, the bifurcation angle can be calculated precisely with CT, which offers the operator clear guidance on whether bifurcation strategies more suited to open angles, such as T-and-protrusion, are optimal, or closed-angle techniques, such as the double kissing (DK) crush technique or culotte techniques.

**Fig. 2.**
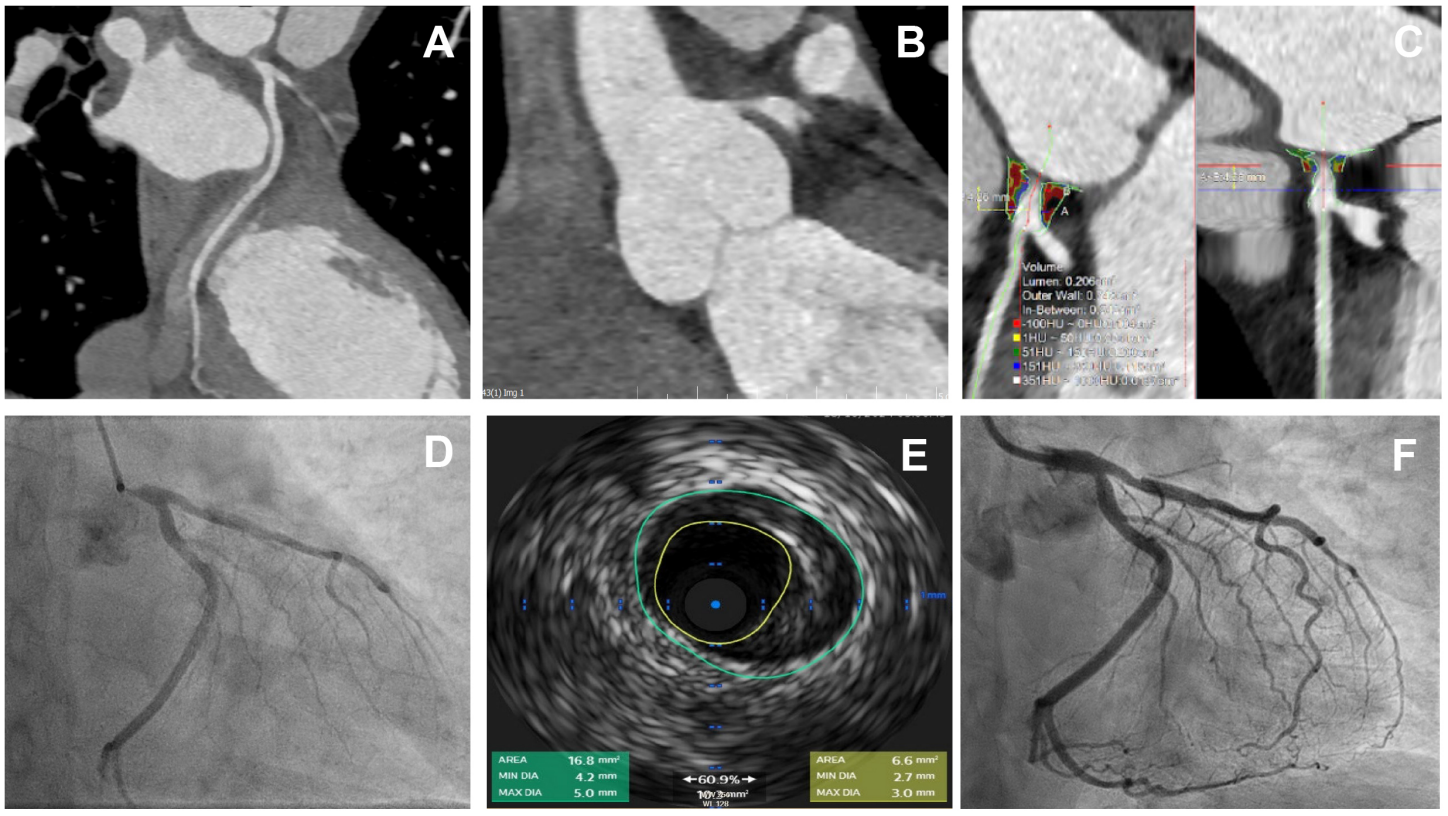
**A case of critical ostial left main stem disease treated with a single drug-eluting stent**. Comparison of intravascular imaging with IVUS and CTCA-derived plaque characterisation. Images represent a case of a 56-year lady with no previous history of coronary artery disease who presented with chest pain. CTCA showed severe ostial LMS disease, which was confirmed by invasive angiography. (A,B) curved multiplanar reconstruction (MPR) demonstrating severe ostial LMS stenosis. (C) Plaque characteristics by TerraRecon software (version 4.4.14, TerraRecon, Durham, USA) show calcified plaque with Hounsfield Unit (HU) range >350, non-calcified plaque (composed primarily of fibrous or lipid components without significant calcification) 30–350 HU. The patient opted for percutaneous coronary intervention (PCI) to LMS rather than surgery. The lesion demonstrated on (D) was predilated with 3 × 12 mm semicompliant ballon. Intravascular ultrasound (IVUS) to proximal LAD back to LMS showed soft plaque and severe ostial stenosis (E). A 4 × 8 mm Onyx stent was implanted and post-dilated with a 5.0 NC balloon with a good final result (F). Moderate proximal LAD stenosis was managed medically.

Given the anatomical variation of the LMS above, CT images of the LMS tapering and length can assist with device sizing in vessel preparation with balloon and stent sizing. These cases are invariably performed with intravascular imaging (IVI) techniques such as intravascular ultrasound (IVUS) or optical coherence tomography (OCT). CT can be used synergistically with IVI as a pre-procedure planning tool (see example Fig. 2). In contrast, IVI is typically interpreted while the patient is on the catheterisation laboratory table, making it less suitable for multidisciplinary team (MDT) discussions and thorough pre-procedural strategy planning. While CT does not replace IVI in this workflow, it provides valuable additional insights, enabling the operator to be better prepared and informed about the likely anatomical challenges before the PCI procedure. CT reconstructions of the anatomy significantly enhance this preparation process, offering a more comprehensive understanding of the patient’s vascular structure.

The assessment of plaque is discussed later in greater detail; however, CT reconstructions also allow for the sizing of devices required in calcified LMS and LMS with high burdens of fibro-atheromatous plaque. Again, when used with IVI, CT provides information on which type of plaque modification techniques will likely be required. A common occurrence is that not all catheterisation laboratories have all techniques available at all times. Thus, CT can direct the team to equip the catheterisation laboratory with the correct devices, which they are likely to need, increasing the efficiency of the cath lab workload.

Finally, CT informs of the dimensions of the aortic root and the take-off of the ostium. Interventional cardiologists sometimes encounter atypical anatomy, such as aberrant take-offs of the LMS coronary ostium. Pre-procedural CT scanning can provide valuable information regarding selecting a guide catheter, thereby avoiding repeated efforts to find a catheter appropriately shaped for the specific patient. Again, choosing a guide catheter with sufficient support and the correct size improves efficiency and simplifies the procedure.

## 4. Plaque Morphology for Vessel Preparation and Calcium Modification

CTCA helps rule out atherosclerosis or detect subclinical plaque. Therefore, it can be used to monitor the progression of CAD with preventive therapy while aiding in risk classification. Conversely, in patients with significant obstruction, CTCA allows evaluation of the burden of plaque disease, lesion length, and plaque composition [30].

CTCA, using the latest-generation scanners, has proven to be an accurate, non-invasive method for assessing and quantifying coronary plaque volume, showing an excellent correlation with IVUS [30]. In the Coronary CT Angiography EvaluatioN For Clinical Outcomes: An InteRnational Multicenter (CONFIRM) registry, which included 12,086 patients undergoing CCTA for suspected coronary artery disease, 3.5% had obstructive unprotected LMS disease [31]. Regarding the prognostic value of plaque composition on all-cause mortality, the registry showed none of the plaque components improved the diagnostic accuracy of the model. Still, the overall burden of calcified plaques improved the prediction [32]. Although a few smaller observational studies found correlations between plaque composition and the outcome, it remains controversial. See the summary of evidence supporting the prognostic value of CT-derived plaque characterisation in Table 2 (Ref. [33, 34, 35, 36, 37, 38, 39, 40, 41, 42, 43, 44, 45]).

**Table 2.** **CTCA-derived plaque characteristics and evidence confirming correlation with intravascular ultrasound (IVUS)**.

Author	Study Type	Population	Number of Patients	LMS	Objective/Outcomes	Key Outcomes	Conclusion/Relevance
Section A: CTCA and correlation with IVUS							
Leber *et al*. (2005) [33]	Prospective	Stable CAD	55 Patients	16 (29%)	Objective: Determine the diagnostic accuracy of CTCA to identify and quantify atherosclerotic coronary lesions in comparison with ICA + IVUS	CTCA allowed the identification of proximal coronary lesions with excellent accuracy. Good correlation with IVUS data	CTCA provided good correlation with IVUS in measuring plaque and luminal area
Boogers *et al*. (2012) [34]	Prospective	Patients who had CTCA and ICA with IVUS for assessment of CAD	51 (103 coronary vessels)	N/S	Objective: Feasibility and accuracy of automated coronary plaque quantification on CTCA using dedicated software with a 3D co-registration algorithm of CT and IVUS data sets	Good correlation between CTCA and IVUS for MLA, Lumen area stenosis, plaque burden, mean plaque burden and remodeling index	Automated quantification of coronary plaque on CT is feasible and offers a good correlation with IVUS
Park *et al*. (2015) [35]	Retrospective	Patients with suspected CAD who underwent Both CTCA, and ICA with IVUS	142 (150 coronary segments)	8 (5.3%)	Objective: To evaluate the diagnostic performance of automated coronary atherosclerotic plaque quantification (QCT) by different users (expert/non-expert/automatic) compared to IVUS	Excellent correlation between CTCA and IVUS in terms of MLA, %AS, %PB, and plaque volume especially with expert analysis	CTCA can offer an accurate assessment of significant lesions and may help guide PCI planning
Munnur *et al*. (2020) [36]	Retrospective	Patients who had CTCA and IVUS for assessment of suspected CAD	27 patients (769 vessel segments analysed)	9 (28%)	Primary Objective: Compare the accuracy of plaque quantification by automated and manual methods on CTCA using IVUS as the reference standard	Manual plaque quantification on CTCA was comparable to IVUS per slice. Excellent association between CTCA high-risk plaque features and IVUS echo-attenuated plaques	CTCA is effective for non-invasive plaque assessment and high-risk feature identification
					Secondary Objective: Assess the association between plaques with features of EA and EL on IVUS with high-risk plaque features on CTCA		
Conte *et al*. (2020) [37]	Retrospective	Patients who underwent CTCA and IVUS	118 (59 in 64s-slice CT, 59 in whole-heart coverage CT)	N/S	Objectives: Evaluate whether last-generation CTCA may improve coronary plaque volume assessment using IVUS as a standard of reference	High correlation for plaque volume quantification by CCTA vs IVUS (higher in whole heart coverage CTCA). Mild Plaque volume overestimation by CTCA (More in 64 slice CT)	CTCA is an accurate non-invasive tool to assess and quantify coronary plaque volume
Thakur *et al*. (2024) [38]	Prospective	Stable CAD	58	58	Objective: Assess whether quantitative CTCA measures could assist clinicians in making LMS revascularization decisions when compared with IVUS measurements as gold standard	CTCA-derived MLA and MLD had a strong correlation with IVUS. CTCA-derived MLA cut-off <8.29 mm^2^ showed the greatest utility for predicting the need for further assessment	CTCA can provide a good utility in assessing LMS disease
Section B: CTCA derived Coronary Plaque characteristics							
Nakazawa *et al*. (2008) [39]	Prospective	CAD diagnosed on CTCA before PCI	51	3	Objectives: Investigate the impact of CT density values in culprit lesions on the occurrence of transient no-reflow during PCI	In the 9 Patients who had transient no-reflow. Low CT density value and NR signs were more frequent	LAP and NR sign are a predictor of transient no-reflow during PCI
Motoyama *et al*. (2015) [40]	Retrospective	Known CAD detected by CTCA	3158	(4 out of 88 events)	Objective: Evaluate whether plaque characteristics by CTA predict the mid-term likelihood of ACS	ACS occurred in 88 patients. ACS more common with APCs and significant stenosis	CTCA-derived APCs and plaque progression are both independent factor for ACS
Nadjiri *et al*. (2016) [41]	Retrospective	Suspected CAD	1168	N/S	Objectives: Assess the incremental prognostic value of quantitative plaque characterization beyond established CT risk scores	LAPV, TPV, PR, and the presence of the napkin-ring sign are predictors of MACE independently of clinical risk presentation. Low risk if CTCA negative	APCs such as LAPV, TPV PR, and NR sign are independent predictors of MACE
					Primary endpoints: CVD, MACE, revascularization		
Sekimoto *et al*. (2016) [42]	Retrospective	Stable CAD	116 (168 lesions)	5	Objectives: Asses if high per-lesion coronary calcium scores are an independent predictor for the addition of rotablation during PCI	Target lesion length ≥20 mm and a diameter stenosis ≥74% on ICA, as well as a target lesion length ≥32 mm and a presence of a per-lesion calcium score ≥453 on CTCA were the primary factors associated with rotablation. Also grade 5 calcification	CTCA may help in predicting the need for a calcium modification strategy during PCI
Feuchtner *et al*. (2017) [43]	Prospective	Stable CAD	1469	N/S	Objective: To assess the prognostic value of CTCA for prediction of MACE over a long-term follow-up period	The prognosis is excellent if CTCA is negative and worsens with increasing non-calcifying plaque component. LAP and NR sign are the most powerful MACE predictors	APCs such as LAP and NR sign are strong predictors of MACE
					Primary endpoint: MACE		
					Secondary endpoint: Coronary revascularization (PCI or CABG)		
Williams *et al*. (2019) [44]	(Post-hoc analysis of SCOT-HEART trial)	CTCA arm of the trial	1769 (26,535 segments)	60 (3.4%)	Objective: Investigate the prognostic implications of APCs in patients with suspected CAD	APCs and overall calcified plaque burden confer an increased risk of CVD, and nonfatal MI	CTCA can be used to assess adverse plaque characteristics leading to MACE
CRISP-CT (2018) [45]	Prospective	Patients undergoing CTCA	3912 (1872 in the derivation cohort, 2040 in validation cohort)	None	Objectives: Assess the predictive value of the perivascular FAI for the two primary endpoints of all-cause mortality and cardiac mortality	The perivascular FAI enhances cardiac risk prediction and re-stratification	CTCA-derived FAI may predict risk of MACE and guide primary prevention

The overview of studies investigating the correlation between CTCA and IVUS for assessing coronary plaque characteristics and using CTCA-derived plaque analysis. The findings highlight the potential CTCA’s role in identifying high-risk plaque features and its utility as a non-invasive surrogate for IVUS, particularly in assessing LMS disease and other complex lesions.

Plaque morphology, such as ‘nodular’ versus ‘smooth’ calcifications, can influence the effectiveness of drug-coated balloons in drug delivery, with smoother surfaces allowing for more uniform distribution [46]. Calcification in LMS poses challenges, such as difficulty in device delivery and incomplete stent apposition, which are associated with worse outcomes [47]. The application of such technologies to LMS remains an evolving area requiring targeted trials. Hence, CTCA’s capability to identify high-risk plaque features and provide comprehensive plaque characterisation positions it as a potential alternative to IVI in the future, as supported by recent randomised controlled trials (RCTs), including the ISCHEMIA trial [48]. In the ISCHEMIA trial, CCTA was pivotal in the non-invasive assessment of coronary anatomy before randomisation [48]. CCTA was utilised to exclude patients with significant LMS stenosis (≥50%) and to identify individuals without obstructive CAD. This approach ensured that participants met the anatomical eligibility criteria, enhancing the trial’s safety and the validity of its findings. However, the clinical significance of incorporating these plaque characteristics into decision-making for LMS PCI has yet to be validated in clinical trials.

Considering the role of CTCA in the characterisation of plaque composition, distinguishing between calcified, fibrous, and lipid-rich plaques, which is critical for LMS PCI planning strategies, such as using adjunctive devices like rotational atherectomy for heavily calcified lesions, it is hard to dismiss its value. Accurate evaluation of plaque volume, lesion length, and vessel size is also essential for optimal stent selection, ensuring appropriate coverage and apposition, often achieved through intravascular imaging modalities like IVUS or OCT (Fig. 2). CTCA’s ability to visualise proximal and distal reference segments has already been shown to enhance precise stent placement [49]. Additionally, CTCA can detect anatomy that may lead to plaque-related complications, such as calcific nodules or thrombus, necessitating pre-treatment or additional devices to mitigate procedural risks. These insights may drive tailored procedural and post-PCI management strategies, further expanding CTCA’s utility in LMS PCI.

## 5. CTCA-Derived Functional Assessments

CTFFR offers additional value in understanding coronary vasculature in CTCA (Table 1) [30]. The reasons for this are similar to ICA and FFR derived from an invasive measurement of trans-lesional pressure with a guidewire. FFR itself is valuable in evaluating coronary artery disease, and FFR derived from CT has been shown to correlate well [50]. Therefore, CTFFR can be added to clinical practice to guide decision-making. Several studies have shown the clinical validation of CTFFR in patients with obstructive coronary artery disease, with a similar cut-off to invasive measurements [30, 50]. In addition, CTFFR has been deemed accurate in complex cases in patients with multi-vessel coronary artery disease. Finally, CTFFR has likely economic benefits, being cost-effective and significantly reducing the overall healthcare cost for a patient undergoing investigation for obstructive coronary disease [51].

The calculation of the CTFFR involves integrating anatomical and physiological data derived from CCTA to assess CAD severity using functional assessment derived from anatomical studies using a computation method. This calculation typically starts with segmenting the coronary arteries from CTCA images to reconstruct a 3D model of the coronary vasculature. Computational fluid dynamics (CFD) techniques are then applied to simulate blood flow and pressure under rest and hyperaemic conditions, mimicking stress states. The model calculates pressure gradients across stenotic lesions using boundary conditions derived from patient-specific data, such as heart rate and blood pressure. The CTFFR value is expressed as the ratio of the distal coronary pressure (post-stenosis) to the aortic pressure (pre-stenosis), offering a non-invasive approximation of the invasive FFR measurement. Advances in machine learning have also introduced fast CTFFR techniques that use image-based algorithms to bypass the computational intensity of traditional CFD, providing comparable accuracy. The clinical utility of CTFFR lies in its ability to identify hemodynamically significant stenoses using the same CTCA dataset, guiding decisions for revascularisation while avoiding invasive procedures. The recently developed PCI Planner, which integrates CTFFR to support the planning and evaluation of various percutaneous intervention strategies in complex coronary artery disease, holds promise as a valuable tool in the future preparation for LMS PCI [52].

Further studies are needed to support the use of CTFFR in patients with LMS disease in a similar way as the use of invasively measured FFR in patients with LMS disease. However, some data have suggested the utility of CTFFR for evaluating LMS stenoses, and the short-term outcomes in patients with CT-derived FFR >0.80 are favourable [53].

## 6. Simulation of LMS PCI and Prediction of Functional Results of PCI

As stated above, predictive tools such as virtual FFR (vFFR) and machine learning models are increasingly being applied to assess PCI outcomes. vFFR, derived from CT imaging or CFD simulations, offers a non-invasive method to predict post-PCI haemodynamics (see the summary of evidence in Table 1). Machine learning algorithms, trained on large datasets, provide predictive insights into restenosis rates, stent thrombosis risk, and long-term survival. These tools are evolving to include LMS-specific scenarios, addressing this critical lesion’s unique anatomical and flow challenges. LMS PCI significantly impacts coronary blood flow due to its crucial role in supplying a significant portion of the myocardium. Restoring normal haemodynamics in LMS stenosis often requires precise stent deployment to ensure optimal luminal gain and minimal flow disturbance. Studies have shown that incomplete stent expansion or malposition in LMS can result in adverse flow dynamics, increasing the risk of restenosis and thrombosis. Studies suggest that assessing post-PCI FFR in LMS can improve the accuracy of predicting functional recovery and long-term outcomes [54, 55].

## 7. Assessment of Post-PCI Results

**Fig. 3.**
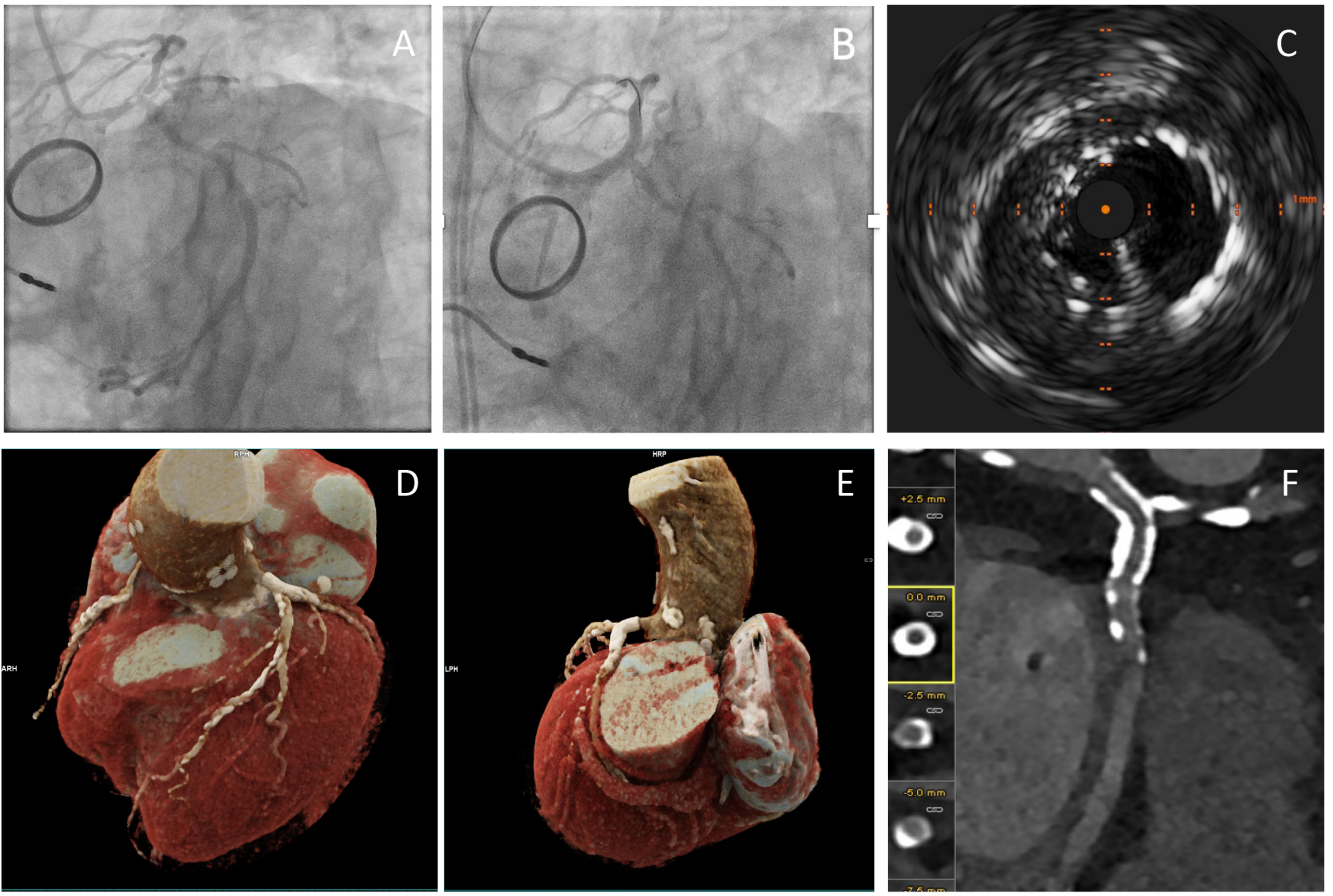
**Comprehensive Coronary Artery Assessment Using Photon-Counting Computed Tomography (PCCT) following a PCI to LMS**. (A,B) present invasive angiography before and after LMS stent insertion. (C) shows IVUS post-PCI to LMS. (D,E) show high-resolution, three-dimensional visualisation of the coronary arteries. It confirms normal origins and right dominance. It shows widely patent metallic stents in the LMS segment and the presence of extensive mixed calcified and non-calcified atherosclerotic plaques, with apparent high-grade stenosis in the mid-LAD. Proximally patent stents are visualised in the left circumference artery (F). It is an example of how PCCT may improve the diagnostic accuracy post-PCI to the LMS in complex coronary artery disease. The high sensitivity and specificity for detecting significant stenoses and stent patency highlight the utility of PTCT. This approach is particularly advantageous in anatomically challenging cases, such as those involving LMS interventions. Images were acquired with the NAEOTOM Alpha® photon-counting CT scanner (Siemens, Germany).

Studies have demonstrated that CCTA can reliably detect in-stent restenosis [17, 56, 57]; hence, CTCA is an invaluable non-invasive tool for assessing outcomes following PCI, particularly in complex procedures, including LMS PCI. It enables detailed evaluation of stent positioning, expansion, and apposition, ensuring optimal placement and functionality. CTCA is particularly useful for detecting in-stent restenosis by identifying luminal narrowing caused by neointimal hyperplasia. CTCA provides a precise, non-invasive approach for assessing in-stent restenosis in stented unprotected LMS, minimising the risks associated with invasive catheterisation (Fig. 3). It reconstructs stented vessels with limited contrast dye and a low radiation dose [58]. Moreover, CTCA facilitates the identification of post-procedural complications, such as stent fractures, malposition, and thrombus formation, while also allowing for the assessment of residual plaque morphology and vessel remodelling. Advanced applications, such as CTFFR, can enhance its utility by providing functional insights into coronary flow dynamics pre and post-PCI in LMS PCI [59]. Thus, CTCA offers a comprehensive approach for anatomical and physiological follow-up in LMS PCI as a non-invasive alternative to invasive angiography [53].

## 8. CT Angiography in Patients With Diabetes

Diabetes mellitus is associated with poorer clinical outcomes in patients undergoing any revascularisation procedure, which is a direct result of the high burden of atherosclerotic plaque, longer lesion lengths, and abnormal neointimal proliferation following stenting [60]. Although the choice of revascularisation strategy for patients with diabetes, three-vessel disease, and/or LMS disease remains a subject of discussion between cardiologists and cardiac surgeons within the Heart Team multidisciplinary meetings, the extent of disease in patients with diabetes favours the surgical option. In this context, CTCA offers a valuable non-invasive alternative to traditional invasive angiography, particularly for patients with diabetes with LMS involvement. Given that coronary artery bypass grafting (CABG) is often the preferred treatment option for this population, and they are at higher risk of kidney disease, minimising contrast exposure becomes crucial. Additionally, patients with diabetes frequently present with multivessel disease and are less likely to experience typical chest pain, further underscoring the importance of non-invasive assessment tools like CTCA in guiding optimal management. A proof-of-concept study proposed the potential role of CTCA as a single, non-invasive, and comprehensive imaging modality for planning CABG [61]. This finding was subsequently supported by a larger clinical trial, which assessed the feasibility of using CTCA for CABG planning and demonstrated an acceptable safety profile in a carefully selected cohort of patients with complex CAD [62]. Thus, CTCA should be considered the preferred imaging modality for assessing CAD in high-risk diabetic patients, owing to its non-invasive nature, comprehensive diagnostic capabilities, and ability to minimise contrast exposure while facilitating optimal intervention planning.

## 9. Future Directions

To establish CTCA as the preferred imaging modality for LMS assessment, further research is needed to validate its ability to replace ICA and intravascular imaging techniques such as OCT and IVUS. Robust comparative studies should evaluate its efficacy in guiding LMS revascularisation and ensuring outcomes equivalent to or better than those achieved with invasive techniques. Such validation is critical to position CTCA as a reliable, non-invasive alternative in clinical practice. Further health economic evaluations are also required to establish the cost-effectiveness of CTCA compared to invasive imaging techniques, particularly in resource-limited healthcare systems. Efforts to improve accessibility and streamline CTCA workflows will be critical for broader adoption.

Advancements in CTCA technology, including improved spatial and temporal resolution and the increasing availability of photon-counting CT (PCCT) scanners, will undoubtedly enhance the diagnostic accuracy of CTCA in managing LMS disease. The potential of PCCT to revolutionise LMS imaging lies in its ability to produce high-resolution, low-dose, and spectrally enriched images with accurate plaque characterisation analysis (see examples in Fig. 3) [63]. PCCT offers superior differentiation of plaque components and calcifications, with reduced calcium blooming compared to energy-integrating detector CT images, resulting in more accurate luminal stenosis estimates [63, 64]. These capabilities are critical for determining appropriate interventional strategies, as they enable better visualisation of plaque morphology and calcification alongside assessments of the functional significance of stenosis, which is believed to be comparable if not more accurate than interventional imaging techniques.

Integrating CTFFR and leveraging artificial intelligence (AI) further enhances the precision and utility of CTCA in LMS disease evaluation [65]. AI algorithms can automate the detection and quantification of stenosis, perform risk stratification, and provide decision support for revascularisation strategies. Incorporating AI into clinical workflows can enhance diagnostic efficiency and support more precise, individualised patient management [66]. Research into AI-driven approaches could also uncover novel ways to analyse complex imaging datasets, further advancing the utility of CTCA in LMS assessment given the multitude of predictors of LMS PCI outcomes.

Future exploration should aim to develop and standardise CTCA acquisition, post-processing, and reporting protocols specifically for LMS assessment. These protocols should include guidelines on contrast usage, imaging parameters, and interpretation criteria. Standardisation would ensure consistency and reliability across institutions and clinical practices, facilitating the broader adoption of CTCA.

As evidence supporting the utility of CTCA in LMS revascularisation grows, it will be essential to update clinical practice guidelines to reflect its role as a first-line imaging modality to facilitate the integration of CTCA into routine care. Future studies should prioritise defining the role of CTCA in high-risk patient populations, such as individuals with diabetes, chronic kidney disease, peripheral vascular disease or multivessel disease, where non-invasive imaging has clear advantages over traditional invasive methods due to their elevated risk of complications from invasive procedures and complex clinical profiles. The transformative potential of CTCA in LMS disease management underscores the need for continued innovation, multidisciplinary research, and collaborative efforts to realise its full clinical value.

## 10. Conclusions

CTCA is emerging as a pivotal non-invasive imaging modality, offering detailed anatomical and functional assessments that significantly enhance the diagnosis, planning, and follow-up of LMS percutaneous coronary intervention and has been recently shown to be a feasible imaging option to plan coronary artery bypass surgery. Recent advancements, including 3D vessel reconstructions, CTFFR, and integration of AI algorithms, have further expanded its capabilities, providing valuable insights into plaque morphology, lesion complexity, and bifurcation anatomy. These features are particularly advantageous for guiding LMS PCI, where pre-procedural planning and precise intervention strategies are critical due to the complexity and high peri-procedural risks associated with LMS disease. Despite these advancements, further research is necessary to establish CTCA as a first-line imaging modality capable of replacing invasive ICA in assessing LMS disease. Large, prospective studies are required to evaluate its diagnostic accuracy, cost-effectiveness, and clinical impact in guiding PCI and assessing post-PCI outcomes, particularly in complex cases and high-risk groups such as diabetic patients. In conclusion, while CTCA shows tremendous promise as a transformative tool in LMS disease management, ongoing innovation and multidisciplinary research are essential to fully realise its potential and define its role as a non-invasive alternative to traditional invasive imaging techniques.

## Abbreviations

CAD, Coronary artery disease; CCS, Chronic coronary syndrome; CT, Computed tomography; CTCA, Computed tomography coronary angiography; CTFFR, Computed tomography derived fractional flow reserve; ICA, Invasive coronary angiography; IVI, Intravascular imaging; IVL, Intravascular lithotripsy; IVUS, Intravascular ultrasound; LAD, Left anterior descending artery; LCx, Left circumflex artery; LMS, Left main stem; MPR, Multiplanar reconstructions; PCI, Percutaneous coronary intervention; 3D, Three-dimensional.
